# Computerized clinical decision support systems for prescribing in primary care: main characteristics and implementation impact—protocol of an evidence and gap map

**DOI:** 10.1186/s13643-022-02161-6

**Published:** 2022-12-29

**Authors:** Héctor Acosta-García, Ingrid Ferrer-López, Juan Ruano-Ruiz, Bernardo Santos-Ramos, Teresa Molina-López

**Affiliations:** 1 Pharmacy Service, University Hospital Virgen del Rocio, Seville, Spain; 2 Primary Care Pharmacist Service, Sevilla Primary Care District, Seville, Spain; 3 Dermatology Service, IMIBIC/Reina Sofía University Hospital/University of Cordoba, Cordoba, Spain

**Keywords:** Evidence and gap maps, Computerized clinical decision support systems, Electronic prescribing decision support system, Primary healthcare

## Abstract

**Background:**

Computerized clinical decision support systems are used by clinicians at the point of care to improve quality of healthcare processes (prescribing error prevention, adherence to clinical guidelines, etc.) and clinical outcomes (preventive, therapeutic, and diagnostics). Attempts to summarize results of computerized clinical decision support systems to support prescription in primary care have been challenging, and most systematic reviews and meta-analyses failed due to an extremely high degree of heterogeneity present among the included primary studies. The aim of our study will be to synthesize the evidence, considering all methodological factors that could explain these differences, and build an evidence and gap map to identify important remaining research questions.

**Methods:**

A literature search will be conducted from January 2010 onwards in MEDLINE, Embase, the Cochrane Library, and Web of Science databases. Two reviewers will independently screen all citations, full text, and abstract data. The study methodological quality and risk of bias will be appraised using appropriate tools if applicable. A flow diagram with the screened studies will be presented, and all included studies will be displayed using interactive evidence and gap maps. Results will be reported in accordance with recommendations from the Campbell Collaboration on the development of evidence and gap maps.

**Discussion:**

Evidence behind computerized clinical decision support systems to support prescription use in primary care has so far been difficult to be synthesized. Evidence and gap maps represent an innovative approach that has emerged and is increasingly being used to address a broader research question, where multiple types of intervention and outcomes reported may be evaluated. Broad inclusion criteria have been chosen with regard to study designs, in order to collect all available information. Regarding the limitations, we will only include English and Spanish language studies from the last 10 years, we will not perform a grey literature search, and we will not carry out a meta-analysis due to the predictable heterogeneity of available studies.

**Systematic review registration:**

This study is registered in Open Science Framework https://bit.ly/2RqKrWp

**Supplementary Information:**

The online version contains supplementary material available at 10.1186/s13643-022-02161-6.

## Background

Computerized clinical decision support systems (CDSS) match the characteristics of patients to a computerized medical knowledge base and provide recommendations to clinicians for consideration. CDSS emerged five decades ago as powerful tools for guiding clinical practice and has shown a rapid evolution since then. A recent overview on the use of CDSS in medicine has addressed risks, benefits, functions, and advantages of CDSS in the areas of patient safety, clinical management, cost containment, diagnostics support, and patient-facing decision support [1]. The development of CDSS-P began in hospital settings [2], but soon they also became relevant in primary care, usually linked to the global adoption of electronic health record (EHR) in this setting [3, 4].

Several systematic reviews about CDSS for prescribing (CDSS-P) in hospital settings describe a wide focus of these systems ranging from assisting clinicians in drug selection and dosing suggestions, alerting potential adverse drug reactions and drug allergies, identifying duplication of therapy, and advising change prescribing in accordance with guidelines. They also identified a wide range of levels of complexity and interactions between the information in the patient’s clinical record and the information relating to the prescribed medication [5–13]. However, meta-analyses of CDSS-P about clinical effectiveness have proved to be challenging [14]. Literature about CDSS-P in the context of primary healthcare is extensive, but most of the overviews about effectiveness and safety of these systems have failed in the efforts to synthesize data quantitatively using meta-analysis s due to a high heterogeneity in baseline characteristics, outcome measures, and statistical analysis of primary studies [15, 16]. In other cases, reviews have focused on summarizing the evidence of CDSS-P on very specific contexts such as on population subgroups [17], application area [18], care setting [19], type of medication [20–23], or studied outcomes [23].

As far as we know, there are no systematic reviews either synthesizing the most recent available evidence about CDSS-P in primary care or describing in detail main features of these system. To use of CDSS in practice, it is important to understand the basic requirements of these systems: how they operate, their complexity, extent to which they interact with the data in clinical records or they integrate artificial intelligence technologies, their learning potential or interoperability, capability to use new sources of data, i.e. big data, or their effect on the providers who use them, patient outcomes, or costs.

Finally, evidence gap maps (EGMs) are an interactive visual tool designed to provide an overview of the existing evidence on a topic to promote evidence-informed policy and prioritize future research [24]. The use of EGMs facilitates evidence-based decision-making since it produces a better summary of the evidence obtained by a systematic review. EGMs also identify the evidence gaps that can be the basis for future research in a field and may identify potential areas where a specific development is needed and appropriate.

## Methods

### Aims/objectives

The aim of our systematic review and EGM is as follows: first, systematically identify and describe CDSS-P in primary care, their functional and operational characteristic, the evidence about their effectiveness, their implementation process, and the planned evaluation, and secondly, to inform developers and decision-makers for about design, implementation, evaluation, and maintenance of CDSS-P and future research.

### Study design

This protocol has been developed following the PRISMA-P [25] and PRISMA-ScR [26] guidelines, using the methodology described in the Joanna Briggs Institute Reviewer’s Manual [27] as well as those recently published relating to EGMs [24].

### Protocol and registration

This study has been registered in Open Science Framework (https://osf.io/g3mdy/?view_only=54c2497bc6f04ad8b1cf549dc7ac6299).

### Patient and public involvement

We did not involve patients or the public in the conduction of this protocol.

### Eligibility criteria

Eligible studies should meet the following criteria: English or Spanish language; healthcare provided by the prescribing professionals — the end-users of the CDSS-P being investigated — should happen in a primary care setting; population will include both home-dwelling patients as well as nursing home patients; the studies could be focused either on the description of the systems, in whole or in part, or their utility, effectiveness or impact; and primary and descriptive studies will be included, along with intervention studies that compare the support system to the usual clinical practice.

Studies that do not include a description of the functioning of the system will be excluded from the EGM. Nonscientific reviews and opinion articles will be excluded.

### Research questions

Research questions are as follows:

- Which CDSS-P in primary care have been described in the scientific literature?
- What are their main characteristics, from an operational viewpoint?
- What impacts have they shown in improving prescribing and health outcomes? Do they achieve the objectives for which they were designed?
- What types of studies have described these systems and how many studies of each type have been found about each system?
- What gaps in knowledge have been identified regarding these systems?

### Information sources

MEDLINE — through the OvidSP platform —, Embase, The Cochrane Library and Web of Science database will be searched from January 2010 to the present day:

### Search strategy

The search strategies have been developed by one member of the research team, who has extensive experience in structured searches and handling information sources. Additional file 1:  Databases Search strategies details the complete strategies used in the databases (MEDLINE, Embase, the Cochrane Library, and Web of Science) screened and the number of articles after applying each filter.

If a systematic review were identified, the studies included in it would be analysed to incorporate them into the search. In order to identify additional studies, a new search will be performed using the reference lists of all selected reports and articles for identification of additional relevant studies. Furthermore, before finalizing the data extraction process, a search update will be performed in order to identify studies that may have been published between the search closing date and the end of the data extraction process.

Through the systematic search, after excluding duplicates, 810 references were identified. Reference and full text for all documents identified through the literature search will be imported into Mendeley® reference management tool and Excel® spreadsheet, where they will be compiled in an ad hoc table, classifying them as included, excluded, or duplicated.

### Selection of sources of evidence

Two reviewers working independently will screen the title and abstract to decide for inclusion. Disagreement will be resolved through discussion and consensus. In order to increase the consistency between the reviewers, both of them will examine the same first hundred publications. Full manuscript of potentially relevant citations will be obtained and the criteria re-applied.

The list of articles that will be selected at first but then rejected after a full-text revision will be included in a specific Annex, accompanying the final publication.

### Data charting

A data charting form will be jointly developed by two reviewers to determine which variables to extract. A pilot test will be carried out with five studies, and the chosen variables were included in a .csv file. The two reviewers independently will chart the data, discuss the results, and continuously update the data charting form in an iterative process. Data extraction from the selected studies will be carried out by four members of the research team, working in pairs and using a predefined empty table. Any discrepancies that may arise will be resolved by each pair through discussion and consensus. In the case that no agreement is reached, a third researcher will be included in the discussion, and, ultimately, a vote will be carried out.

### Data items

The following data will be collected from each study: the date and geographic area where the study was carried out (Europe, USA, ROW), the type of healthcare organization in which the system was developed, the study type and objectives, the size of the included population, the aim of the system being evaluated, the point of comparison it is being evaluated against, the evaluation variables, and the authors’ main results and conclusions. Furthermore, data will be collected to describe the system being evaluated in each study: a general description of its purpose, its level of complexity, whether the intervention carried out in the study was single- or multi-component, if it focuses on medications or pathologies, if the system is independent or is integrated into a specific electronic prescribing system, whether it is considered an intelligent system, and the main data sources it interacts with (prescriptions, diagnoses, laboratory data, functional testing, etc.).

### Critical appraisal of individual sources of evidence

Quality appraisal and risk-of-bias assessment are optional but not mandatory steps in scoping reviews or EGMs and are not often conducted [26]. So, if we finally decide to carry it out, we will describe which methods and tools will be implemented. The rationale for this decision and the reasons for choosing the pertinent assessment tools will be given.

### Synthesis of results and visualization

We do not expect to find data relevant for conducting a meta-analysis. All information will be categorized, and a narrative and qualitative evidence synthesis will be conducted. Tables and figures (i.e. bubble plot) will be used to display the evidence landscape and to elucidate clusters and gaps. We have developed a conceptual model of system categories through a process of debate and consensus taking into account the characteristics described in the literature cited in the introduction, regarding prescribing support systems in hospitals [5–14] and primary care [15–22].

### Tables

According to the conceptual model, results will be grouped by the following: (a) application focus (medications, pathologies, or prescription adequacy to protocols or guidelines); (b) system functionality; and (c) level of system complexity (grouped into five categories from lesser to greater complexity). The description of the system will also include the level of integration in the electronic prescribing system (in the event that it is integrated, the electronic prescribing system in which it is integrated will also be described), the databases with which it interacts, and whether it can be considered an intelligent system.

Every study will be described in terms of the following: (1) geographic area where it was implemented; (2) type of healthcare organization that developed the support system; (3) objectives, design, and population (number of observed patients, distinguishing between the intervention and control groups, when relevant; (4) characteristics of the identified CDSS-P; (6) comparator/control; (7) outcomes; (8) study’s main conclusions; and (9) methodological quality and risk of bias, if applicable.

When appropriate, data related with the type of study, number of subjects involved, and outcome results will be included. The tables will also specify whether any cost-effectiveness analysis was identified. Time-course and geographic differences will be analysed.

The results of the comprehensive search will be presented using a PRISMA flow diagram. Finally, a table summarizing study methodological quality and risk of bias of each study will be provided as supplement.

### Evidence and gap maps

EGMs will be produced including only primary studies that assess intervention effectiveness. An interactive table will be designed to provide an overview of the existing evidence and to graphically highlight the evidence gaps and the time that it will show a summary of the studies. Colums will display system complexity and study outcomes (health outcomes, use of health resources, potentially inadequate prescribing/medication errors avoided, and acceptance), and rows will display the purpose or context of the decision support system (these will be defined based on search results).

Additional dimensions will be added using different colours, shapes, and sizes to plot studies on the map. Each table cell will show studies sharing design and quality features represented as separate symbols. If we finally perform a methodological quality evaluation, a traffic light colour-coding system will be used to display the results about the risk of bias of included studies as green, yellow, and red corresponding to high, medium, and low confidence findings, respectively. The colour transparency effects, symbol directions (up: favours the new intervention; down: favours the standard system), and colour intensity (colour: significant *p* < 0.05; grey: not significant *p* > = 0.05) of each plot will represent the magnitude, direction, and significance of interventional effects. A series of pop-up brief text messages will be displayed when the user scrolls over each cell. Finally, the map will allow the user to filter the information and display only certain subgroups of studies, for example filtering by study design, geographic location, result or direction of the effect.

For the presentation of the maps, the information relating to the identified systems and their characteristics will be entered in a dynamic and interactive platform. The data will be organized in columns according to the results in health outcomes identified in the studies and the level of complexity of the support systems and in rows according to the subcategory of the system’s purpose. The cells in the table will contain different geometric shapes according to the study type and different colours according to the results variables and quality of the study, and the cell’s size will be proportional to the number of patients included in the total of the included studies. The location in the cell will give information on the direction of the effect. As the maps will be interactive, the user will be able to click on each of the figures and obtain a list of the relevant studies. From this list, they can then click on each of the studies to access a URL of the study in question. An example of EGMs is shown in Additional file 2: Example of Evidence and Gaps Maps.

The data model will be designed in such a way that it exhaustively compiles all of the possibilities existing to this day and will allow for the detection of gaps in knowledge for each of them. The identified knowledge gaps will be laid out in specific tables.

## Discussion

EGMs represent an innovative approach to the presentation of available evidence to potential users, decision-makers, and the scientific community, including both funding entities and researchers, who need to decide how best to allocate their financing or their research projects, respectively.

As there is not a generally accepted guideline to carry out an EGM, we have followed, firstly, two PRISMA guidelines, the guideline for scoping reviews [26] and the guideline for protocols elaboration [25], and the methodology described in the Joanna Briggs Institute Reviewer’s Manual [27]. Secondly, we have also integrated the recently published recommendations on the development of EGMs [24].

Searching for original studies, instead of systematic reviews, will enable access to original and detailed descriptions of the CDSS-P, which is one of the aims of this project. Broad inclusion criteria have been chosen with regard to study designs, in order to collect all available information about CDSS-P in primary care. The classification according to their purpose and level of complexity among other characteristics will allow representing a gap map that could be easily used for investigators when defining further research projects.

One of the limitations of the study is that it only includes studies published in English and Spanish, which could lead to the exclusion of information concerning specific systems that have been written about on a local level. Similarly, there will be no search of the grey literature, which could again lead to the loss of corporate information from organizations that have not published scientific papers. The restriction of the literature search to the last 10 years could also be a limitation, although we consider that extending the search beyond this period of time would not allow us to discover tools that are still valid today as these are a kind of tools that evolves rapidly and soon becomes obsolete Indeed, the speed of the development of these tools can lead to another limitation, which is the potential loss of certain publications made between the closing date of the study and the publication of the article. Lastly, due to the expected heterogeneity in the interventions, designs, results, and measurement systems, our project will not carry out a meta-analysis of the effectiveness or cost-effectiveness of the systems.

## Supplementary Information

**Additional file 1.** Databases Search strategies.

**Additional file 2.** Example of Evidence and Gaps Maps.

**Additional file 3.** Preferred Reporting Items for Systematic reviews and Meta-Analyses extension for Scoping Reviews (PRISMA-ScR) Checklist.

## Data Availability

All data generated or analysed during the EGM will be included in the future published article (and its supplementary information files).

## Abbreviations

CDSS: Computerized clinical decision support systems
CDSS-P: Computerized clinical decision support systems for prescribing
EGM: Evidence and gap map
EGMs: Evidence and gap maps
EHR: Electronic health record
ROW: Rest of World
