# Supplementary material for: Computerized clinical decision support systems for prescribing in primary care: main characteristics and implementation impact—protocol of an evidence and gap map
Source: Syst Rev. 2022 Dec 29;11:283. doi: 10.1186/s13643-022-02161-6 (PMC9798565; doi:10.1186/s13643-022-02161-6)
Supplement: Supplementary file 1 — Additional file 1. Databases Search strategies. [file 13643_2022_2161_MOESM1_ESM.pdf]

Annex 1.

Databases Search strategies:

Medline:

Date of search: 03-Apr-21

1. (Primary Health Care or Nursing Homes or Ambulatory Care or Ambulatory Care Facilities).sh……...169194

2. (Primary Care or Primary health care or Primary healthcare or Primary medical care or Family practi* or Family medicine or General practi* or Family physician* or Family Doctor* or Nurse Practition* or nursing home*).mp. [mp=title, abstract, original title, name of substance word, subject heading word, floating sub-heading word, keyword heading word, organism supplementary concept word, protocol supplementary concept word, rare disease supplementary concept word, unique identifier, synonyms]…………………………………….…309374

3. 1 or 2………..362845

4. (Medical Order Entry Systems or Medical Records Systems, Computerized or User-Computer Interface or Drug Prescriptions).sh……………………………..83553

5. (electronic medical record system* or medical order entry system* or medical information system* or user-computer interface or drug prescription* or electronic prescription or electronic prescribing or e prescribing or e-prescribing or e-prescription or e prescription or cpoe or computerized provider order entry or computerized physician order entry or computer-assisted drug therap* or automated medical record system or computerized medical record* or records, computerized medical or medical records, computerized or prescription* or prescribing*).mp. [mp=title, abstract, original title, name of substance word, subject heading word, floating sub-heading word, keyword heading word, organism supplementary concept word, protocol supplementary concept word, rare disease supplementary concept word, unique identifier, synonyms]……………………………………………………………………………..163817

6. 4 or 5………..179698

7. (Decision Support Systems, Clinical or Decision Support Techniques or Decision Support Systems, Management or Clinical Decision-Making or Decision Making, Organizational or Drug Therapy, Computer-Assisted or Clinical Pharmacy Information Systems).sh…………………..49341

8. (computer assisted drug therapy or clinical decision support system* or clinical decision support or support, clinical decision or Clinical Decision Support Systems or Clinical Decision Support or Clinical Decision Supports or Decision Supports, Clinical or Support, Clinical Decision or Supports, Clinical Decision or Decision Support, Clinical or Decision Support Technique or Technique, Decision Support or Techniques, Decision Support or Decision Support Technics or Decision Support Technic or Technic, Decision Support or Technics, Decision Support or Models, Decision Support or Decision Support Modelo r Decision Support Models or Model, Decision

Support or Decision Modeling or Modeling, Decision or Decision Aids or Aid, Decision or Aids, Decision or Decision Aid or Decision Analysis or Analyses, Decision or Decision Analyses or Analysis, Decision or Clinical Decision Making or Decision-Making, Clinical or Medical Decision-Making or Decision-Making, Medical or Medical Decision Making or Drug Therapy, Computer Assisted or Therapy, Computer-Assisted Drug or Computer-Assisted Drug Therapies or Drug Therapies, Computer-Assisted or Therapies, Computer-Assisted Drug or Therapy, Computer Assisted Drug or Computer-Assisted Drug Therapy or Computer Assisted Drug Therapy or Protocol Drug Therapy, Computer-Assisted or Protocol Drug Therapy, Computer Assisted).mp. [mp=title, abstract, original title, name of substance word, subject heading word, floating sub-heading word, keyword heading word, organism supplementary concept word, protocol supplementary concept word, rare disease supplementary concept word, unique identifier, synonyms]…………………………………………………………………………….37100

9. 7 or 8……….68949

10. 3 and 6 and 9……..827

11. limit 10 to (adaptive clinical trial or case reports or clinical study or clinical trial, all or clinical trial, phase i or clinical trial, phase ii or clinical trial, phase iii or clinical trial, phase iv or clinical trial or comparative study or controlled clinical trial or multicenter study or observational study or pragmatic clinical trial or randomized controlled trial)………………….215

Embase:

Date of search: 04-Apr-21

#1. 'primary health care'/exp OR 'nursing home'/exp OR 'ambulatory care'/exp OR 'outpatient department'/exp……………………………………………………………………………………………………….332,615

#2. 'primary care':ab,ti OR 'primary health care':ab,ti OR 'primary healthcare':ab,ti OR 'primary medical care':ab,ti OR 'family practi*':ab,ti OR 'family medicine':ab,ti OR 'general practi*':ab,ti OR 'family physician*':ab,ti OR 'family doctor*':ab,ti OR 'nurse practition*':ab,ti OR 'nursing home*':ab,ti…….342561

#3. #1 OR #2…….522826

#4. 'electronic medical record system'/de OR 'physician order entry system'/de OR 'computer interface'/de OR 'prescription'/de OR 'electronic prescribing'/de…………………………………..231103

#5. 'electronic medical record system*':ab,ti OR 'medical order entry system*':ab,ti OR 'medical information system*':ab,ti OR 'user-computer interface':ab,ti OR 'drug prescription*':ab,ti OR 'electronic prescription':ab,ti OR 'electronic prescribing':ab,ti OR 'e prescribing':ab,ti OR 'e-prescribing':ab,ti OR 'e-prescription':ab,ti OR 'e prescription':ab,ti OR 'cpoe':ab,ti OR 'computerized provider order entry':ab,ti OR 'computerized physician order entry':ab,ti OR 'computer-assisted drug therap*':ab,ti OR 'automated medical record system':ab,ti OR 'computerized medical record*':ab,ti OR 'records, computerized medical':ab,ti

OR 'medical records, computerized':ab,ti OR 'prescription*':ab,ti OR 'prescribing*':ab,ti……………………………………………………………………………………………………….214622

#6. #4 OR #6…….……….317532

#7. 'clinical decision support system'/de OR 'decision support system'/de OR 'clinical decision making'/de OR 'computer assisted drug therapy'/de OR 'medical information system'/de………..89536

#8.'clinical decision support system*':ab,ti OR 'clinical decision support systems':ab,ti OR 'clinical decision support':ab,ti OR 'clinical decision supports':ab,ti OR 'decision supports, clinical':ab,ti OR 'support, clinical decision':ab,ti OR 'supports, clinical decision':ab,ti OR 'decision support, clinical':ab,ti OR 'decision support technique':ab,ti OR 'technique, decision support':ab,ti OR 'techniques, decision support':ab,ti OR 'decision support technics':ab,ti OR 'decision support technic':ab,ti OR 'technic, decision support':ab,ti OR 'technics, decision support':ab,ti OR 'models, decision support':ab,ti OR 'decision support model':ab,ti OR 'decision support models':ab,ti OR 'model, decision support':ab,ti OR 'decision modeling':ab,ti OR 'modeling, decision':ab,ti OR 'decision aids':ab,ti OR 'aid, decision':ab,ti OR 'aids, decision':ab,ti OR 'decision aid':ab,ti OR 'decision analysis':ab,ti OR 'analyses, decision':ab,ti OR 'decision analyses':ab,ti OR 'analysis, decision':ab,ti OR 'clinical decision making':ab,ti OR 'decision-making, clinical':ab,ti OR 'medical decision-making':ab,ti OR 'decision-making, medical':ab,ti OR 'medical decision making':ab,ti OR 'drug therapy, computer assisted':ab,ti OR 'therapy, computer-assisted drug':ab,ti OR 'computer-assisted drug therapies':ab,ti OR 'drug therapies, computer-assisted':ab,ti OR 'therapies, computer-assisted drug':ab,ti OR 'therapy, computer assisted drug':ab,ti OR 'computer-assisted drug therapy':ab,ti OR 'computer assisted drug therapy':ab,ti OR 'protocol drug therapy, computer-assisted':ab,ti OR 'protocol drug therapy, computer assisted':ab,ti…………..46230

#9. #7 OR #8………115883

#10. #3 AND #8 AND #9…………………………………………………………………………………………..….1329

#11. #10 AND ('clinical study'/de OR 'clinical trial'/de OR 'cohort analysis'/de OR 'comparative effectiveness'/de OR 'comparative study'/de OR 'control group'/de OR 'controlled clinical trial'/de OR 'controlled study'/de OR 'cross sectional study'/de OR 'intervention study'/de OR 'major clinical study'/de OR 'medical record review'/de OR 'multicenter study'/de OR 'observational study'/de OR 'prospective study'/de OR 'randomized controlled trial'/de OR 'retrospective study'/de)………………………………………………………………………………………………...580

#12. #11 AND (2010:py OR 2011:py OR 2012:py OR 2013:py OR 2014:py OR 2015:py OR 2016:py OR 2017:py OR 2018:py OR 2019:py OR 2020:py OR 2021:py)……………………………… 393

The Cochrane Library

Date of search: 08-Apr-21

#1 MeSH descriptor: [Primary Health Care] explode all trees……………………………………7035

#2 MeSH descriptor: [Nursing Homes] explode all trees……………………………………………..1314

#3 MeSH descriptor: [Ambulatory Care] explode all trees………………………………………..3596

#4 MeSH descriptor: [Ambulatory Care Facilities] explode all trees……………………….....1814

#5 MeSH descriptor: [Outpatient Clinics, Hospital] explode all trees………………………….648

#6 MeSH descriptor: [Ambulatory Care Information Systems] explode all trees……………..25

#7 #1 or #2 or #3 or #4 or #5 or #6…………………………………………………………………….13078

#8 (“primary care” OR “primary health care” OR “primary healthcare” OR “primary medical care” OR “family practi*” OR “family medicine” OR “general practi*” OR “family physician*” OR “family doctor*” OR “nurse practition*” OR “nursing home*”):ti,ab,kw…24169

#9 #7 or #8……………………………………………………………………………………………………..31893

#10 MeSH descriptor: [Ambulatory Care Information Systems] explode all trees……………..25

#11 MeSH descriptor: [Medical Records Systems, Computerized] explode all trees………..576

#12 MeSH descriptor: [Drug Prescriptions] explode all trees…………………………………………878

#13 #10 or #11 or #12………………………………………………………………………………………..1452

#14 ("electronic medical record system*" or "medical order entry system*" or "medical information system*" or "user-computer interface or drug prescription*" or "electronic prescription" or "electronic prescribing" or "e prescribing" or "e-prescribing" or "e-prescription" or "e prescription" or "cpoe" or "computerized provider order entry" or "computerized physician order entry" or "computer-assisted drug therap*" or "automated medical record system" or "computerized medical record*" or "records, computerized medical" or "medical records, computerized" or "prescription*" or "prescribing*"):ti,ab,kw…………………………………………………………………………………………15451

#15 #13 or #14…………………………………………………………………………………………………..16406

#16 MeSH descriptor: [Decision Support Systems, Clinical] explode all trees……………….352

#17 MeSH descriptor: [Decision Support Techniques] explode all trees………………………2409

#18 MeSH descriptor: [Clinical Decision-Making] explode all trees………………………………..204

#19 MeSH descriptor: [Decision Making, Computer-Assisted] explode all trees……………4955

#20 MeSH descriptor: [Drug Therapy, Computer-Assisted] explode all trees…………………..147

#21 MeSH descriptor: [Decision Making, Organizational] explode all trees………………………42

#22 #16 or #17 or #18# or #19 or #20 or #21………………………………………………………………7759

#23 (“clinical decision support system*” OR “clinical decision support systems” OR “clinical decision support” OR “clinical decision supports” OR “decision supports, clinical” OR “support, clinical decision” OR “supports, clinical decision” OR “decision support, clinical” OR “decision support technique” OR “technique, decision support” OR “techniques, decision support” OR “decision support technics” OR “decision support technic” OR “technic, decision support” OR “technics, decision support” OR “models, decision support” OR “decision support model” OR “decision support models” OR “model, decision support” OR “decision modeling” OR “modeling, decision” OR “decision aids” OR “aid, decision” OR “aids, decision” OR “decision aid” OR “decision analysis” OR “analyses, decision” OR “decision analyses” OR “analysis, decision” OR “clinical decision making” OR “decision-making, clinical” OR “medical decision-making” OR “decision-making, medical” OR “medical decision making” OR “drug therapy, computer assisted” OR “therapy, computer-assisted drug” OR “computer-assisted drug therapies” OR “drug therapies, computer-assisted” OR “therapies, computer-assisted drug” OR “therapy, computer assisted drug” OR “computer-assisted drug therapy” OR “computer assisted drug therapy” OR “protocol drug therapy, computer-assisted” OR “protocol drug therapy, computer assisted”):ti,ab,kw……………………………………………………………………………..5695

#24 #22 or #23……………………………………………………………………………………………………12582

#25 #9 and #15 and #24 in Trials………………………………………………………………………………225

Web of science

Date of search: 09-Apr-21

#1. TI=(“primary care” OR “primary health care” OR “primary healthcare” OR “primary medical care” OR “family practi*” OR “family medicine” OR “general practi*” OR “family physician*” OR “family doctor*” OR “nurse practition*” OR “nursing home*”)…………………………………116359

#2. AB=(“primary care” OR “primary health care” OR “primary healthcare” OR “primary medical care” OR “family practi*” OR “family medicine” OR “general practi*” OR “family physician*” OR “family doctor*” OR “nurse practition*” OR “nursing home*”)………………162676

#3. #1 OR #2………223821

#4. TI=("electronic medical record system*" or "medical order entry system*" or "medical information system*" or "user-computer interface or drug prescription*" or "electronic prescription" or "electronic prescribing" or "e prescribing" or "e-prescribing" or "e-prescription" or "e prescription" or "cpoe" or "computerized provider order entry" or "computerized physician order entry" or "computer-assisted drug therap*" or "automated medical record system" or "computerized medical record*" or "records, computerized medical" or "medical records, computerized" or "prescription*" or "prescribing*")…………44320

#5. AB=("electronic medical record system*" or "medical order entry system*" or "medical information system*" or "user-computer interface or drug prescription*" or "electronic prescription" or "electronic prescribing" or "e prescribing" or "e-prescribing" or "e-

prescription" or "e prescription" or "cpoe" or "computerized provider order entry" or "computerized physician order entry" or "computer-assisted drug therap*" or "automated medical record system" or "computerized medical record*" or "records, computerized medical" or "medical records, computerized" or "prescription*" or "prescribing*")……….111682

#6. #4 OR #5……..137656

#7. TI=("clinical decision support system*" OR "clinical decision support systems" OR "clinical decision support" OR "clinical decision supports" OR "decision supports, clinical" OR "support, clinical decision" OR "supports, clinical decision" OR "decision support, clinical" OR "decision support technique" OR "technique, decision support" OR "techniques, decision support" OR "decision support technics" OR "decision support technic" OR "technic, decision support" OR "technics, decision support" OR "models, decision support" OR "decision support model" OR "decision support models" OR "model, decision support" OR "decision modeling" OR "modeling, decision" OR "decision aids" OR "aid, decision" OR "aids, decision" OR "decision aid" OR "decision analysis" OR "analyses, decision" OR "decision analyses" OR "analysis, decision" OR "clinical decision making" OR "decision-making, clinical" OR "medical decision-making" OR "decision-making, medical" OR "medical decision making" OR "drug therapy, computer assisted" OR "therapy, computer-assisted drug" OR "computer-assisted drug therapies" OR "drug therapies, computer-assisted" OR "therapies, computer-assisted drug" OR "therapy, computer assisted drug" OR "computer-assisted drug therapy" OR "computer assisted drug therapy" OR "protocol drug therapy, computer-assisted" OR "protocol drug therapy, computer assisted")……………………………………………………………………………………………13468

#8. AB=("clinical decision support system*" OR "clinical decision support systems" OR "clinical decision support" OR "clinical decision supports" OR "decision supports, clinical" OR "support, clinical decision" OR "supports, clinical decision" OR "decision support, clinical" OR "decision support technique" OR "technique, decision support" OR "techniques, decision support" OR "decision support technics" OR "decision support technic" OR "technic, decision support" OR "technics, decision support" OR "models, decision support" OR "decision support model" OR "decision support models" OR "model, decision support" OR "decision modeling" OR "modeling, decision" OR "decision aids" OR "aid, decision" OR "aids, decision" OR "decision aid" OR "decision analysis" OR "analyses, decision" OR "decision analyses" OR "analysis, decision" OR "clinical decision making" OR "decision-making, clinical" OR "medical decision-making" OR "decision-making, medical" OR "medical decision making" OR "drug therapy, computer assisted" OR "therapy, computer-assisted drug" OR "computer-assisted drug therapies" OR "drug therapies, computer-assisted" OR "therapies, computer-assisted drug" OR "therapy, computer assisted drug" OR "computer-assisted drug therapy" OR "computer assisted drug therapy" OR "protocol drug therapy, computer-assisted" OR "protocol drug therapy, computer assisted")……………………………………………………………………………………………...34246

#9. #7 OR #8……..42022

#10. #9 AND #6 AND #3………………………………………………………………………………………………..214

#11. #9 AND #6 AND #3. Filtered by: TYPE OF DOCUMENT (ARTICLE)……………………………….……191

#12. #9 AND #6 AND #3. Filtered by: TYPE OF DOCUMENT (ARTICLE) AND LANGUAJE: (ENGLISH)………..189
