# Supplementary material for: Computerized clinical decision support systems for prescribing in primary care: main characteristics and implementation impact—protocol of an evidence and gap map
Source: Syst Rev. 2022 Dec 29;11:283. doi: 10.1186/s13643-022-02161-6 (PMC9798565; doi:10.1186/s13643-022-02161-6)
Supplement: Supplementary file 2 — Additional file 2. Example of Evidence and Gaps Maps. [file 13643_2022_2161_MOESM2_ESM.pdf]

Annex 2.

Example of Evidence and Gaps Maps:

			Outcomes				
			Acceptance/Use	Potentially Inappropiate Prescription (PIP)	Health	Economic / Use of resources	Adherence to guidelines
INTERVENTIONS	DRUGS	Usual / Maximum / Minimum dose	●				
		Usual posology	●				
		Drug and food intake	●				
		Treatment length alert	●		●●●●●●		
		Alert on administration route	●				
		Duplicate drugs		●			
		Interaction checker			●		
		Contraindications				●	
		Anticholinergic load alert	●				
		Deprescription		●			
